# A Cross Cultural Understanding of Diverse Dementia Journeys

**DOI:** 10.1002/alz.087722

**Published:** 2025-01-09

**Authors:** Mary Chi Michael

**Affiliations:** ^1^ Otsuka America Pharmaceutical Inc., Princeton, NJ USA

## Abstract

**Background:**

Over the past 3 years, the Global Council on Alzheimer’s Disease (GCAD) has conducted research on lived experience and care partner journeys. Specifically, this research has focused on the experiences of individuals from historically underrepresented populations, including LGBTQ+, Black, Hispanic/Latino, and Asian communities. The goal has been to identify how these journeys might diverge across communities, understand various nuances that exist across cultures, and recognize the impact these might have on seeking diagnosis, care, and support.

**Method:**

To understand the experiences of community members living with dementia, we undertook qualitative primary research, complemented by secondary research. Primary research consisted of dozens of one‐on‐one interviews with people living with dementia and their care partners. Secondary research consisted of a review of existing literature on each community’s experiences with dementia.

**Result:**

An output of this work is a visualization that maps and overlays distinct journeys that members of each community experience. These journey maps illustrate how one community’s journeys might overlap and diverge compared to others. The maps capture the challenges each community faces, resources that have been developed, and how care is managed along the disease progression.

**Conclusion:**

This work has revealed many nuances that exist across the dementia journey, including: different manifestations of stigma, bias, medical inequity, unequal access to care and treatment; how care is managed both institutionally and in the home; impacts of the presence or absence of traditional family care structures; and varying availability of resources for living well with dementia. It also revealed areas in which each community is overcoming that stigma as well as opportunities for support and resources. The project concludes with a call to action that outlines how the dementia community can better support people affected by dementia from the underrepresented communities; it also emphasizes the importance of applying culturally competent approaches to understanding these journeys and ultimately providing better care and support.

